# Supplementary material for: Protective Effect of Lactiplantibacillus plantarum subsp. plantarum SC-5 on Dextran Sulfate Sodium—Induced Colitis in Mice
Source: Foods. 2023 Feb 20;12(4):897. doi: 10.3390/foods12040897 (PMC9957050; doi:10.3390/foods12040897)
Supplement: Supplementary file 1 [file foods-12-00897-s001.zip › foods-2150518-supplementary.pdf]

Table S1. DAI score

Score	Percentage of weight loss	Fecal viscosity	Blood stool
0	0	Normal	Negative
1	1-5%	Loose stool	Cambridge blue
2	5-10%	Mucoid stool	Blue
3	10-20%	Dilute liquid stool	Dark blue
4	>20%		Gross blood stool

Table S2. Histopathology score

Score	The number of ulcers	The changes in Epithelial cells	The degree of inflammatory infiltration	The number of lymph nodes
0	0	Normal	None	None
1	1	A small loss of goblet cells	Pericrypt inflammatory infiltrate	1
2	2	Extensive loss of goblet cells	Inflammatory infiltration in muscularis mucosae	2
3	3	Loss of crypts	The muscularis mucosae were generally infiltrated with inflammation and the mucosa was thickened.	3
4	>3	Extensive loss of crypts or Polypoid regeneration	Inflammatory infiltration of the submucosa	>3
